# Prevalence of sexual violence and associated factors among women attending antenatal care in Debre Markos at public health institutions in north-west Ethiopia, 2021

**DOI:** 10.3389/fgwh.2023.1009272

**Published:** 2023-02-20

**Authors:** Marta Yimam Abegaz, Haymanot Alem Muche, Getie Lake Aynalem, Tazeb Alemu Anteneh, Nebyu Solomon Tibebu, Getachew Muluye Gedef, Aregash Sitot Mengstie

**Affiliations:** School of Midwifery Department of Clinical Midwifery, College of Medicine and Health Science, University of Gondar, Gondar, Ethiopia

**Keywords:** sexual violence, pregnant, antenatal care, Debre Markos, Ethiopia

## Abstract

**Background:**

Sexual violence refers to being forced to perform any unwanted sexual act. Due to the negative effects on both the mother and the fetus, sexual assault during pregnancy is a public health concern. Knowing the prevalence of sexual violence during pregnancy helps policymakers understand the extent of the problem and can be considered the first step toward implementing interventions for prevention and treatment. This study was done to determine the prevalence of sexual violence and its associated factors during pregnancy in public hospitals in Debre Markos.

**Methods:**

An institution-based cross-sectional study was conducted among 306 pregnant women in Debre Markos, north west Ethiopia from May 1 to June 30, 2021, 2021. A systematic random sampling procedure was used to select study participants. Data were collected using a structured and interviewer-administered questionnaire, and a pre-test was done. Both bi-variable and multivariable logistic regression analyses were undertaken to identify significantly associated variables with sexual violence. The adjusted odds ratio (AOR) with its 95% confidence interval (CI) at a *p*-value of ≤ 0.05 was used to claim statistical association.

**Results:**

There were 304 interviewed respondents with a response rate of 99.3%. In this study, the overall prevalence of sexual violence among pregnant mothers during the current pregnancy was 19.4%. A husband who had no formal education {AOR = 3.48; 95% CI: 1.06, 11.39}, pregnant mothers who had no formal education {AOR =  6.1; 95% CI: 1.50–18.11}, pregnant mothers who had secondary education {AOR = 2.80, 95% CI: 1.15, 6.81}, being a housewife {AOR = 3.87, 95 CI:1.21, 12.37}, and being a governmental employee {AOR = 4.49, 95% CI: 1.22, 16.40} were factors associated with sexual violence at the value of *p* ≤ 0.05.

**Conclusions and recommendations:**

In this study, approximately one-fifth of the study participants experienced sexual violence during their current pregnancy. To reduce this, interventions should focus on the education of women as well as their partner about violence against women and on initiatives to economically empower women.

## Background

Violence against women is a fundamental violation of women's human rights and a significant public health problem. Violence against women ranges from less severe to the most severe forms of violence, and sexual violence is among the most severe forms of violence (1). Sexual violence is defined as any sexual act, trying to gain a sexual act, or other actions directed against a person sexually using pressure, by any person regardless of their relationship to the victim, in any setting (2). Pregnancy is regarded as a socially and culturally regardful period of a woman's life. Despite there being several regulations, rules, and programs addressing violence against women, efforts to address violence specifically during pregnancy are still minimal (3).

Globally, approximately 27% of reproductive-age (15–49 years) partnered women have experienced sexual violence at least once in their lifetime (4). During pregnancy, the prevalence of violence has been reported to be between 4%–29%, which is grossly underreported specially in developing countries (5, 6). Violence against women is a social problem that prevents them from participating fully in social and cultural life (7). Indeed, although Ethiopia has put in place applicable and effective legal and policy provisions to stop violence against women, it is still a major problem in the country (8). A study conducted in Ethiopia showed that in Tigray approximately 15.5%, and, 11.3% in Hulet Ejju Enessie District, of participants experienced sexual violence, which included forced coitus, unwanted sexual intercourse because of fear of their partner, and being forced to do something sexual that is degrading or humiliating to them (9, 10). Women who are exposed to sexual violence are at risk of developing pregnancy-related anxiety and post-partum depression (11). Sexual violence during pregnancy has a high prevalence, has significant adverse health effects on the fetus, and could adversely affect the physical and mental states of the mother as well as increasing the prevalence of complications related to pregnant women such as premature rupture of membrane, acute injuries, eating disorders, dysfunction, sleep disorders, stress disorders, depression, substance abuse, and self-murder (12, 13). Sexual violence during pregnancy can cause adverse fetal and neonatal outgrowth, fetal distress, fetal death, low birth weight, and admission to the neonatal intensive care unit (14). Pregnant women with a history of sexual violence were admitted for hyperemesis, bleeding, and preterm birth (15). Pregnant mothers who have been sexually abused by their partners were less likely to make four or more ANC visits (5). To minimize sexual violence during pregnancy building positive relations between partners is very important (16). Some studies which were conducted in Ethiopia showed that the plan of pregnancy, economic status, level of education, and occupation are factors that affect sexual violence during pregnancy (10, 16, 17). Despite this substantial health burden, previous studies have focused on the prevalence and determinants of sexual violence against women in general. Thus, this study aimed to assess sexual violence among pregnant mothers in public institutions in Debre Markos.

## Methods

### Study area and period

This study was conducted in Debre Markos public health institutions from May 1 to June 30, 2021. Debre Markos is found in the East Gojjam zone, Amhara regional state, located at a distance of 303 kilometers away from Addis Ababa. The town is 265 km from Bahir Dar Debre and has one comprehensive specialized hospital and three public health centers that provide antenatal care (ANC) services.

### Study design and population

A facility-based cross-sectional study was conducted among pregnant women. All pregnant mothers receiving ANC in the public health facilities of Debre Markos were the source population. Pregnant women receiving ANC during the data collection period constituted the study population. All pregnant women receiving ANC at Debre Markos public health institutions during the data collection period were included in the study but pregnant women who are under 18 years and came alone to the health facilities were excluded from the study because it was difficult to get information and consent from them.

### Sample size calculations and sampling procedures

The single population proportion formula was used to determine the sample size with the following assumptions (*n* = z^2^ p (1−p)/d^2^), where z is the normal standard deviation set at 1.96, the confidence level is specified at 95%, the margin of error (d) is 5%, a nonresponse rate of 10%, and a prevalence of sexual violence (p) of 23.7% from a previous study conducted in southeast Oromia (17). The calculated sample size was 306. Proportional allocation to each health facility was done by using the number of pregnant mothers who received ANC in that public health institution based on the number of women who received ANC in monthly reports before data collection. After that systematic random sampling procedure was used to select ANC followers. The sampling interval (K) was gained by dividing the expected total number of monthly ANC service users 1,090 (N) by the number sample size 306 (n) at each data collection site. Every 3rd pregnant mother was selected till the required sample size was achieved.

### Data collection procedure and measurement tool

The questionnaires were prepared in English and then translated into the local language (Amharic) and back to English to sustain their reliability. The questionnaire includes sociodemographic characteristics (age, educational status, religion, residence, and economic status) and reproductive health characteristics (the plan of pregnancy, the decidedness of pregnancy, the age of the first pregnancy, and several ANC follow-ups). The outcome variable (sexual violence) was measured using 3- item questions which were about forced sexual intercourse (sexual intercourse without the consent of the woman), unwanted sexual intercourse because of fear of their husband, and having an unusual type of sexual intercourse that hurts them. Pregnant women reporting at least one act of sexual violence during their current pregnancy were considered to have experienced sexual violence. The pre-test was done with 5% of the pregnant women who had an ANC follow-up outside the study setting to check the wording. Data were collected by five midwives who were BSc degree holders. The training was given to the data collectors and supervisors for two days regarding the general aim of the research by the principal investigator. The proper categorization and coding of the data, as well as the completeness and consistency of the collected data, were checked daily during data collection by the supervisor and authors.

### Data processing and analysis

The collected data were coded, checked manually, entered into Epi-data version 4.1, and then exported into SPSS version 25 for analysis. The result of the uni-variable analysis (descriptive results) was presented as frequencies and percentages. The Chi-square assumption was checked before bivariable analysis. Variables that have a *p*-value ≤ 0.2 were selected for multivariable analysis. The multivariable analysis was performed in the logistic regression up on controlling for the possible confounding factors. Hosmer Lemeshow tests were conducted to test model goodness of fit at value of 0.6. The AOR with its 95% CI and a *p*-value of ≤ 0.05 were used to state statistical association with the outcome variable.

### Ethical approval

Ethical clearance was obtained from the ethical review committee of the School of Midwifery on behalf of the Institutional Review Board (IRB) of the University of Gondar. An official permission letter was obtained from the Debre Markos health office. Informed consent was taken from each study participant after a detailed clarification of the purpose, risks, and benefits of the study. However, written parental consent was given for pregnant girls below the legal age of 18 years. To secure the confidentiality and privacy of participants, the anonymity of patient information was implemented by avoiding personal identifiers and using coding and data locks.

## Results

### Socio-demographic characteristics

There were 304 interviewed respondents with a response rate of 99.3%. Most of the study participants 291 (95.75%) were orthodox in religion and 284 (93.4%) were urban dwellers. Regarding educational level, 137 (45.1%) were in college and above and 38 (12.5%) were private employees. For more than half of the study participants, 184 (60.5%), their monthly income was >3,000 ETB (Table 1).

**Table 1:** socio-demographic characteristics of pregnant women attending ANC at Debre Markos in a public institution of Debre Markos, north-west, Ethiopia, 2021 (*n* = 304).

Variable	Category	Frequency (*n*)	Percent (%)
Age (Years)	15–24	83	27.3
	25–34	187	61.5
	35–49	34	11.2
Religion	Orthodox	291	95.7
	Muslim	9	3
	Protestant	4	1.3
Marital status	Single	11	3.6
	Married	293	96.4
Residence	Urban	284	93.4
	Rural	20	6.6
Educational level of women	No formal education	27	8.9
	Primary education (1–8)	56	18.4
	Secondary education (9–12)	84	27.6
	College and above	137	45.1
Occupation of women	House wife	126	41.4
	Private employee	38	12.5
	Governmental employee	87	28.6
	Merchant	53	17.7
Educational level of husband	No formal education	28	9.2
	Primary education (1–8)	36	11.8
	Secondary education (9–12)	78	25.7
	College and above	151	49.7
Husband occupation	Farmer	8	2.6
	Government employer	134	44.1
	Private employer	97	31.9
	Merchant	54	17.8
Family size	≤3	235	77.3
	≥4	69	22.7
Monthly income	≤3000	120	39.5
	>3000	184	60.5

### Reproductive health characteristics

Of the study participants, 181 (59.5%) were multigravida and 57 respondents (18.8%) had a history of obstetric complications. Of these, 26 (45.5%) had a history of abortion. Regarding pregnancy status, 4 (1.3%) of the pregnancies were unplanned and unwanted (Table 2).

**Table 2:** Reproductive health characteristics pregnant women attending ANC at Debre Markos in a public institution of Debre Markos, north-west, Ethiopia, 2021 (*n* = 304).

Variable	Category	Frequency	Percent %
Gravidity	Primigravida	123	40.5
	Multigravida	181	59.5
Age of first pregnancy	<18	23	7.6
	≥18	281	92.4
History of obstetric complication	Yes	57	18.8
	No	124	40.8
Types of obstetric complication	Abortion	26	45.5
	Still birth	8	14
	IUFD	13	4.3
	Pregnancy induced hypertension	10	17.5
	Yes	26	8.6
History cesarean delivery	No	155	51
	Planned and wanted	258	84.9
Status of current pregnancy	Unplanned and unwanted	4	1.3
	Unplanned but wanted	42	13.8
Period of gestation	Frist trimester	59	19.4
	Second trimester	120	39.5
	Third trimester	125	41.1
ANC follow up	1–3	241	79.3
	≥4	63	20.7

### Prevalence of sexual violence among study participants

Among the 304 interviewed pregnant women, 59 (19.4%) experienced sexual violence. Of those, forced sexual intercourse (8.9%), unwanted sexual intercourse because of fear of the husband (7.2%), and having an unusual type of sexual intercourse that hurt the woman (6.6%) were reported.

### Factors associated with sexual violence

In the binary logistic regression, age, educational levels of the women, occupations of the women, husbands' education, monthly income, family size, and gravidity were associated factors. In the multiple logistic regression, the educational levels of the women, occupations of the women, and the educational level of the husband were significantly associated with sexual violence during pregnancy. Pregnant women whose husbands had no formal education were 3 times more likely to experience sexual violence during current pregnancy (AOR = 3.48; 95% CI: 1.06, 11.39] than pregnant mothers whose husbands attended college and above. The odds of sexual violence among pregnant mothers who had no formal education were 6 times {AOR = 6.1; 95% CI: 1.50–18.11} higher than those women who attended college and above education. In addition, Pregnant women who were in secondary education were 3 (AOR = 2.8, 95% CI: 1.15, 6.81) times more likely to experience sexual violence compared to those women who attended college and above education. Lastly, sexual violence was 4 times {AOR = 3.87, 95% CI: 1.21, 12.37} and 4 times {AOR = 4.49, 95% CI: 1.22, 16.40) more likely to occur among those participants whose occupations were housewife and governmental employee, respectively (Table 3).

**Table 3:** Factors associated with sexual violence among pregnant women attending ANC at Debre Markos in public institution of, Debre Markos, Ethiopia, 2021 (*n* = 304).

variable		Sexual violence		COR with 95% CI	AOR with 95% CI
		Yes	No		
Age	15–24	21	62	6.13 (1.359–27.688)	4.24 (0.69–26.08)
	25–34	36	151	3.56 (0.813–15.56)	5.14 (0.88–30.07)
	35–49	2	32	**1.00**	**1.00**
Educational level of women	No formal education	**7**	**20**	2.47 (0.91–6.71)	6.31 (1.51–18.11)**
	Primary education	13	43	2.13 (0.96–4.76)	2.04 (0.73–5.66)
	Secondary education	22	62	2.50 (1.24–5.06)	2.8 (1.15–6.81)**
	College and above	17	120	**1.00**	**1.00**
Occupation of women	House wife	30	96	3.83 (1.28–11.4)	3.87 (1.21–12.37)*
	Private employee	10	28	4.37 (1.25–1525**)**	4.95 (0.65–12.2**)**
	Governmental employee	15	72	2.55 (0.79–8.15)	4.49 (1.22–16.40)**
	merchant	4	49	**1.00**	**1.00**
Educational level of husband	No formal education	7	20	0.76 (0.24–2.39)	4.40 (1.02–18.62)*
	Primary education	13	43	1.31 (0.54–3.19)	1.95 (0.57–6.70)
	Secondary education	22	62	1.38 (0.70–2.70)	1.76 (0.66–4.65)
	College and above	17	120	**1.00**	**1.00**
Occupation of husband	Farmer	1	7	0.71 (0.08–6.54)	0.22 (0.19–2.61)
	Private employer	28	69	2.03 (0.88–4.70)	1.31 (0.51–3.70)
	Governmental employer	19	115	0.83 (0.35–1.96)	1.54 (0.477–5.05)
	Merchant	9	45	**1.00**	**1.00**
Monthly income	≤3000	33	87	2.31 (1.30–4.10)	1.92 (0.85–4.34)
	>3000	26	158	**1.00**	**1.00**
Family size	**<**3	50	185	0.26 (0.10–0.68)	1.46 (0.58–3.65)
	≥4	9	60	**1.00**	**1.00**
Gravidity	Primigravida	31	92	0.32 (0.18–0.58	1.57 (0.75–3.30)
	Multigravida	28	153	**1.00**	**1.00**

1 = Reference category.

* *P* ≤ 0.05.

** *P* ≤ 0.01.

## Discussion

In this cross-sectional study, the overall prevalence of sexual violence was found to be 19.4%, associated with the educational levels of the women, occupations of the women, and educational level of a husband. This result (19.4%) is similar to another finding from Debre Markos (19.8%) (18). But the finding in this study is higher than studies conducted in Tigray (15.5%) (10), Gondar (7.6%) (16), Harar (3.7%) (19), North West Ethiopia (14.8%) (20), South Sweden (15.5%) (20), Kathmandu (17.3%) (3), and Rwanda (9.7%) (21). This discrepancy may be due to differences in the study period, different sample sizes, and different study designs. Another justification might be that there was an emergency or outbreak of war which increased sexual violence during pregnancy (22). Another explanation for this discrepancy might be the difference in the study population. This study included pregnant women at all trimesters but the study was done in Kathmandu during the third trimester of pregnancy. However, the result in this study is lower than the findings in Oromia (23.7%) (17), Abbay Chomen (30.2%) (23), and Iran (28%) (13). The variation could also be due to the difference in the study subjects, study design, and a discrepancy in the duration of the study period. The study done in Abbay Chomen was community-based and included all reproductive age groups whereas this study included only pregnant women at ANC visits. The initiation of ANC visits promotes healthy sexuality which might reduce sexual violence. Pregnant mothers who had no formal education were 6 times more likely to experience sexual violence than study participants who attended college and above. This outcome is supported by studies done in Oromia (17), Hulet Ejju Enessie (9), Debre Markos (18), and Kathmandu (3). A possible reason could be that educated women have knowledge about sexual and reproductive health rights, which includes a right to make choices that govern their bodies, free of shame and intimidation (24). This was especially the case for women who had attended college and above. Pregnant women who were in secondary education were 3 times more likely to experience sexual violence compared to women who attended college and above. The explanation may be low educational status of women had no power to protect the violence. Women who were housewives were 4 times more likely to experience sexual violence than women who were merchants. This is consistent with the findings in Gondar (16) and Debre Markos (18). A possible explanation might be that in Ethiopia most housewives did not complete their higher education and were kept in their homes. So, they depended on and were abused by their husbands. On the other hand, pregnant mothers who were government employees were 4 times more likely to experience sexual violence compared to merchants. A possible explanation may be the close contact between governmental employees with their colleagues and their bosses may expose them to sexual violence. Sexual violence during pregnancy was 3 times more likely to be reported among pregnant women whose husbands had no formal education. This finding is supported by studies done in Abay Chomen (23) and Debre Markos (18). This might be because educated husbands hold more positive attitudes toward their wives and respect their reproductive rights. However, uneducated husbands may regard the traditional acceptance of violence against their wives as a custom (25).

### Limitations of the study

In some variable measurements, such as forced sexual intercourse, there might be social desirability bias and cultural barriers to disclosing sensitive issues to third parties. Due to the sensitive nature of sexual violence and the cultural barrier of disclosing to third parties, this would lead to underreporting. The other limitation is that some pregnant mothers came in for ANC in early pregnancy but experienced sexual violence later in their pregnancy.

## Conclusion and recommendation

In this study, one-fifth of the study participants experienced sexual violence during pregnancy. The educational status of the woman, the occupational of the woman, and the educational status of her husband were significantly associated with sexual violence during pregnancy. Efforts to reduce sexual violence during pregnancy include increasing awareness about reproductive rights and interventions should focus on the education of women, as well as their partners, about violence against women and on economically empowering women. It is better to conduct community-based interventions for pregnant mothers who are the most at risk, i.e., those that do not attend ANC follow-ups, and conduct further qualitative studies that cover a wider setting for different areas of sexual violence.

## Data Availability

The datasets presented in this study can be found in online repositories. The names of the repository/repositories and accession number(s) can be found in the article/Supplementary Material.
